# Wearable Technologies to Improve Physical Activity, Sedentary Breaks, Training, Sleep, and Safety: The ECSS‐Wearable Technologies Special Interest Group Calls for Interdisciplinary Collaboration

**DOI:** 10.1002/ejsc.70270

**Published:** 2026-11-07

**Authors:** Peter Düking, Marco Giurgiu, Shona Halson, Rene Schilling, Billy Sperlich, Martina Kanning, Birte von Haaren‐Mack, Alexander Ferrauti, Tomas Vetrovsky, Markus Reichert

**Affiliations:** ^1^ Department of Sports Science and Movement Pedagogy Technische Universität Braunschweig Braunschweig Germany; ^2^ Institute of Movement Therapy and Movement‐oriented Prevention and Rehabilitation German Sport University Cologne Cologne Germany; ^3^ School of Behavioural and Health Sciences Australian Catholic University Brisbane Queensland Australia; ^4^ Sports Performance Recovery Injury and New Technologies (SPRINT) Research Centre Australian Catholic University Brisbane Queensland Australia; ^5^ Department of Sport Exercise and Health University of Basel Basel Switzerland; ^6^ Department of Sport Science Julius‐Maximilians‐Universität Würzburg Würzburg Germany; ^7^ Department of Sport Science University of Konstanz Konstanz Germany; ^8^ Independent Researcher; ^9^ Faculty of Sport Science Ruhr University Bochum Bochum Germany; ^10^ Faculty of Physical Education and Sport Charles University Prague Czechia; ^11^ Department of Sport and Exercise Science University of Salzburg Salzburg Austria

## Abstract

Wearable technologies (“wearables”) are lightweight sensor‐based devices that detect, analyze, and transmit physiological, biomechanical or environmental data, and are increasingly used across physical activity, sedentary behavior, athletic training, safety and sleep research. Applications include for example large‐scale physical activity surveillance, ambulatory assessment, and real‐time feedback during athletic training. Despite promising use cases such as heart rate variability‐informed endurance training or gait retraining via biomechanical feedback, critical challenges remain. These include insufficient data quality, inadequately evaluated and inaccessible algorithms, limited interoperability between systems, difficulties translating sensor data into actionable insights, and insufficient wearable technology literacy among users. The Wearable Technologies Special Interest Group of the European College of Sport Science (ECSS‐WT‐SIG) identifies the need for interdisciplinary collaboration to address these challenges. Key priorities include developing regulatory frameworks, promoting standardized and open data infrastructure, improving wearable literacy of users, and conducting rigorous intervention studies under real‐world conditions. The ECSS‐WT‐SIG calls on researchers, industry partners, NGOs, and other stakeholders to collectively advance the scientific and practical application of wearables in physical activity, sedentary behavior, training, sleep, and safety research.

## Highlights

- Wearables support large‐scale, real‐world assessment and feedback across physical activity, sedentary behavior, athletic training, sleep, and safety.
- Key barriers include for example insufficient data quality, limited interoperability, difficulties translating sensor data into actionable insights, and inadequate wearable technology literacy.
- The ECSS‐WT‐SIG calls for interdisciplinary and transdisciplinary collaboration to develop regulatory frameworks, standardized open data infrastructures, educational resources, and rigorous real‐world intervention studies

## Graphical abstract

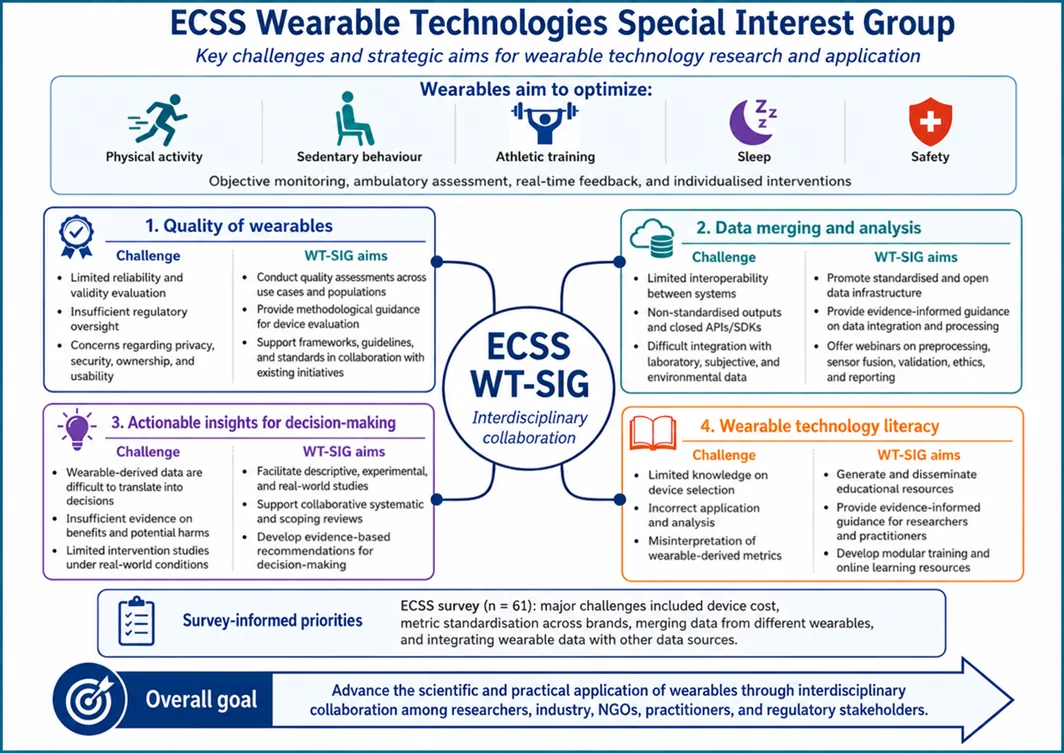

## 1 Introduction

Wearable technologies, commonly referred to as “wearables”, are lightweight sensor‐based devices worn on or near (e.g., in pockets of trousers) the surface of the skin. These devices detect, analyze, and transmit physiological, biomechanical, and/or environmental data to external systems, and in some cases, provide users with immediate biofeedback (Düking et al. 2018). Wearable technologies can be classified according to their anatomical or functional placement, including wrist‐worn devices, ingestible sensors, implantable devices, smart textiles, smart eyewear, hearables, and smart patches.

Wearables have become ubiquitous in modern society and are increasingly employed to optimize physical activity, sedentary behavior, training, safety and sleep practices across both healthy and clinical populations, as well as in research settings.

Since 2016, wearables have consistently ranked among the top trends in the annual global fitness trend reports published by the American College of Sports Medicine, underscoring their growing importance and relevance in the field (McAvoy et al. 2025). Their adoption continues to expand, with approximately 560 million units shipped in 2024 alone, a number projected to grow further in the coming years (Slotta 2025). Wearables are transforming healthcare e.g. via an early disease detection, diagnosis, or treatment of clinical conditions (Babu et al. 2024). Their applications span observational use in large‐scale surveillance studies (e.g., tracking movement behaviors), motivational use in behavioral interventions, and diagnostic support in clinical environments. In athletic populations, wearables are increasingly utilized to assess and provide feedback on relevant training related parameters (e.g., GPS or heart rate related parameters) (Düking et al. 2025), thereby supporting the individualization and optimization of training and performance assessments (Düking, Robertson, et al. 2024; Engel et al. 2026).

Despite rapid technological progress, numerous questions regarding wearables remain unanswered. These are often investigated within isolated disciplinary silos, leading to fragmented research efforts, duplication of work, and missed opportunities for interdisciplinary innovation. This fragmented approach hinders the development of comprehensive integrative solutions. To foster collaboration, promote knowledge exchange, encourage innovation and critical evaluation, and to ultimately advance the field, the European College of Sport Science (ECSS, https://sport‐science.org/) initiated the Wearables Technologies Special Interest Group (ECSS‐WT‐SIG).

Here, the ECSS‐WT‐SIG would like to:

- exemplify the successful application of Wearables in physical activity, sedentary behavior, training, safety, and sleep research
- highlight prominent challenges toward applicability of wearables,
- invite the interdisciplinary sports science community and stakeholders to work jointly with the ECSS‐WT‐SIG on the identified challenges.

### 1.1 Successful Application of Wearables in Physical Activity, Sedentary Behavior, Training, Safety and Sleep Research

#### 1.1.1 Wearables in Physical Activity and Sedentary Behavior Research

Wearables enable the device‐based assessment of a broad range of parameters related to physical activity and sedentary behavior, including but not limited to step count, blood pressure, interstitial glucose levels, and energy expenditure. The most prominent scientific field leveraging wearables for physical activity monitoring is epidemiology, particularly research focused on the prevalence of physical (in)activity and its implications for physical and mental health (Zhu et al., 2024). In this context, large‐scale consortia have been established (e.g., A. Doherty et al. 2017) (https://www.propassconsortium.org; https://ismpb.org/), and several landmark publications have underscored the critical role of physical (in)activity in shaping health outcomes (Ekelund et al. 2019, 2026; WHO 2022).

Beyond epidemiology, psychological research began utilizing wearable technologies over 4 decades ago to investigate human behavior in everyday life contexts (Ebner‐Priemer et al. 2012; Fahrenberg et al. 2007). This tradition has evolved into a methodological framework that integrates tools from psychology, medicine, and computer science to capture real‐time biopsychosocial processes. These approaches, often termed *Ambulatory Assessment*, *Ecological Momentary Assessment*, or *Real‐Time Data Capture*, enable the study of behavior, physiological responses, subjective experiences, and environmental factors within naturalistic settings, thereby increasing ecological validity and supporting real‐time interventions (Ebner‐Priemer et al. 2012) (https://ambulatory‐assessment.org/).

In recent years, driven by advances in digital technologies, Ambulatory Assessment has gained renewed interest in physical activity research (Giurgiu et al. 2025; Rehder et al. 2026; Reichert, Giurgiu et al., 2020). For example, since around 2009, researchers have increasingly applied these methods to examine the dynamic associations between physical activity and affective well‐being in daily life (Giurgiu et al. 2024; Rehder et al. 2026; Timm et al. 2024). The interaction of physical activity and affective well‐being in daily life is of particular importance for both the motivational processes underlying physical activity engagement and for the potential of physical activity as a resilience target for mental disorders. Just recently, an individual participant data meta‐analysis involving 321,345 smartphone‐based well‐being ratings and about 1 million hours of accelerometer‐measured physical activity from  8223 participants across the globe evidenced the importance of physical activity‐affective well‐being relations in human everyday life (Rehder et al. 2026). For example, it revealed a strong connection between physical activity and subjective feelings of energy as a potential target for health behavior change and for the prevention and treatment of affective disorders. Translated to practice, short bouts of physical activity in everyday life, such as climbing stairs or gardening, can make people reliably feel awake and energetic, which may reinforce future physical activity engagement and might help to prevent and treat depression. More recently, Ambulatory Assessment has been combined with laboratory‐based methods to explore neurobiological mechanisms underlying the relationship between everyday physical activity and psychological outcomes. For example, a study combining accelerometry, GPS‐triggered electronic smartphone diaries and structural magnetic resonance imaging found the above‐described connection between physical activity and subjective feelings of energy to be particularly pronounced in neurobiological phenotypes characterized by a low gray matter volume in the subgenual portion of the anterior cingulate cortex (sgACC) (Reichert, Braun, et al. 2020). Given that low gray matter volume in sgACC has been associated with an increased risk for depression, the studies' observation that low‐sgACC individuals suffer in their subjective energy during inactivity but show an increased benefit if being physically active compared to high‐sgACC individuals suggests a risk and resilience mechanism for affective disorders, if this is substantiated by follow‐up studies in clinical samples (Reichert, Braun, et al. 2020). Wearables have also proven valuable in examining how built environments influence physical activity and sedentary behavior. By integrating spatial data, such as maps, environmental features, and travel routes, researchers can assess mobility patterns and identify determinants of physical activity across diverse populations (Yi et al. 2019). Studies have highlighted the importance of geographic and neighborhood contexts, revealing significant intra‐ and inter‐individual variability in physical activity (Moran et al. 2014; Pontin et al. 2022; Smith et al. 2021) The integration of geographic information system (GIS) tools (e.g., to assess light exposure, green and blue spaces, air pollution, and traffic conditions) with wearable‐based (e.g., activity levels) and subjective (e.g., personal preferences) data allows for a better understanding of person‐place interactions (Bollenbach et al. 2022) and highly personalized interventions, such as the development of tailored walking plans for mobility‐limited older adults, and precision medicine approaches in psychiatry (see: https://mobility.dsbg.unibas.ch/de/projekte/mobitec‐routes/) (Reichert et al., 2021; Reichert, Braun, et al. 2020; Tost et al. 2019). Finally, a rapidly growing body of research focuses on how wearables can be employed to promote increases in physical activity. An umbrella review reported that wearable activity trackers, across diverse populations, were associated with approximately 1800 additional steps per day, an increase of 40 minutes of walking per day, and an average reduction in body weight of about 1 kg (Ferguson et al. 2022). However, the review also found that effects on other physiological outcomes (e.g., blood pressure, cholesterol, glycosylated hemoglobin) and psychosocial measures (e.g., quality of life, pain) were generally small and often not statistically significant. Overall, while wearable devices can effectively boost physical activity in the short term, maintaining these behavioral changes over time and achieving meaningful long‐term health outcomes remains a critical challenge for future research (Althoff et al. 2016; Bravata et al. 2007; Finkelstein et al. 2016; M. S. Patel et al. 2017).

#### 1.1.2 Wearables in Athletic Training Research

Wearables are increasingly used in research and applied athletic training to quantify a broad range of internal and external load parameters. A central rationale for their use is that training individualization requires the monitoring, analysis, and contextual interpretation of both external training demands and internal physiological responses (Düking et al. 2021; Schneider et al. 2020). While a great variety of technologies and derived metrics can be obtained, a recent survey (Düking et al. 2025) revealed that heart rate and GPS‐derived metrics are among the most frequently assessed parameters in both research and practice.

In team sports, for example GPS‐based systems (often in combination with inertial measurement units) are commonly used to quantify external load parameters, including positional data, movement velocity, distance covered across different speed zones, accelerations, and decelerations (Cummins et al. 2013; Gómez‐Carmona et al. 2020; Robertson, Duthie, et al. 2023). Beyond load monitoring, these data can also be used to examine tactical behavior during competition, the influence of match phases, and the effects of playing position on individual and collective team behavior. Such information may further support the design of training sessions that more closely replicate the physical and tactical demands of competition, thereby improving the specificity of athlete preparation (Robertson, Duthie, et al. 2023).

In endurance‐based sports, wearable‐derived heart rate data, obtained for example via chest straps or photoplethysmography‐based sensors, are widely used to quantify internal load and to guide training intensity prescription. These data can support for example the assessment of training intensity distribution across different phases of the competitive season (Sperlich et al. 2023).

Beyond these commonly assessed parameters, other parameters assessed by wearables in research around athletic training include for example the application of heart rate variability (HRV) parameters. For example, evidence suggests that, compared with predefined endurance training, HRV‐informed endurance training can induce medium‐sized effects on submaximal physiological parameters, although effects on performance and $\dot{V}$O_2_peak appear to be small and nonsignificant (Düking et al. 2021).

Another application concerns the assessment of biomechanical parameters relevant to running gait analysis (Mason et al. 2023) for example, to examine the effects of fatigue, on running gait, performance aspects or running injuries. While it is beyond the scope of this article to discuss these applications in detail, we refer readers to existing articles (Mason et al. 2023; Van Hooren et al. 2024). Briefly, for example, preliminary evidence indicates that music‐based biofeedback interventions, based on axial peak tibial acceleration data obtained from tri‐axial accelerometers attached to the anteromedial aspects of both tibias, may help runners reduce impact forces during running (Derie et al. 2022). Such approaches appear promising for reducing impact loading, although current evidence is largely derived from controlled laboratory conditions (Derie et al. 2022).

Wearable technologies may also be used to support training individualization in strength training. For example, velocity‐based training approaches use movement velocity to prescribe, monitor, and adjust resistance training intensity and volume. When appropriate devices and parameters are selected, implemented, and interpreted adequately, such approaches may positively affect selected aspects of athletic performance (Balsalobre‐Fernández et al. 2018; Dorrell et al. 2020; Weakley et al. 2020).

#### 1.1.3 Wearables in Sleep Research

Wearable devices (consumer health trackers) have become increasingly popular with both athletes and the general population to monitor sleep and overall health and wellbeing. Worn on the body, these devices are typically non‐invasive and may take the form of a watch, ring, or wrist or arm band. A wearable device is generally not classified as a medical device and therefore not considered as diagnostic tools (Chee et al. 2025).

Raw data are collected by sensors embedded in the wearable device and transferred to a smartphone application that uploads the data to a cloud‐based server. Once uploaded to the cloud, proprietary algorithms analyze the data to provide sleep, activity, and other physiological metrics (Chee et al. 2025). Typically, most wearable devices will use a combination of activity (i.e., movement measured via accelerometery) and autonomic signals (i.e., heart rate (HR) and heart variability (HRV) measured via photoplethysmography (PPG)). Accelerometery is used to measure body movement which decreases with sleep, is further reduced in deep sleep, and is absent during REM sleep in most individuals (Chee et al. 2025). PPG quantifies the lowering of HR and increases in HRV that occurs during sleep onset and across sleep stages, using pulse rate and pulse rate variability as surrogates for HR and HRV, respectively (Chee et al. 2025). Pulse rate and pulse rate variability are calculated by measuring the absorption and reflection of light as it passes through tissue to detect changes in blood volume (Chee et al. 2025). Select devices utilize electrodermal activity sensors to measure changes in skin conductance, which is often inferred as a measure of distal skin temperature change (Chee et al. 2025). It is important to note that performance of wearable devices can vary depending on the quality and number of embedded sensors.

The current evidence supports the validity of the majority of wearables for the assessment of sleep. In a recent and comprehensive study (Miller et al. 2022), compared six consumer health trackers to the reference standard of laboratory‐based polysomnography for sleep staging and electrocardiography for HR and HRV. The devices tested were Apple Watch Series 6, Garmin Forerunner 245 Music, Polar Vantage V, Oura Ring Gen 2, WHOOP 3.0, and Somfit. All six wearables demonstrated high overall agreement (∼86%–89%) with PSG when classifying epochs as sleep or wake (i.e., two‐state sleep). However, accuracy declined when distinguishing specific sleep stages (i.e., multi‐state sleep) including wake, light sleep, deep sleep, and REM sleep, with only moderate agreement observed for all devices (∼50%‐65%). Acceptable agreement with ECG‐based measures was reported for both HR and HRV, particularly for devices sampling continuously over the sleep period (e.g., Oura and WHOOP). While most wearable devices appear accurate for overall sleep detection metrics, their capacity to differentiate different sleep stages is less accurate but likely to continually improve with advances in proprietary algorithms for sleep detection.

Wearable devices provide a unique opportunity for individuals to learn about their sleep patterns and support behavior changes related to sleep. These devices can also provide professionals with access to objective data that may be useful to support the sleep of their patients and clients. For researchers, these devices will become increasingly useful for collecting large amounts of sleep and physiological data in the field. Together, these capabilities position wearable devices as a valuable tool in promoting sleep health across individual, clinical, and research domains.

#### 1.1.4 Wearables for Monitoring Aspects of Safety Related to During Physical Activity and Exercise

Wearable technologies can also be employed for safety monitoring during physical activity and exercise, particularly in contexts where individuals are exposed to increased physiological or biomechanical risks.

Wearables may help (to some extend) to detect early signs of excessive load, fatigue, dehydration, heat stress, falls, immobility, or injury risk, thereby enabling timely interventions before adverse health outcomes occur (Kim et al. 2024; Tseng et al. 2025; Yaldiz et al. 2023). This is relevant not only in athletic settings but also in occupational environments such as construction, agriculture, emergency response, and military service, where individuals often perform strenuous physical work under challenging environmental conditions (Kutilek et al. 2017). For example, inertial sensors integrated into belts, watches, helmets, vests, or smart workwear can detect sudden falls, abnormal posture, prolonged immobility, or unusual movement patterns and automatically alert supervisors, caregivers, or emergency services. When combined with localization systems, such approaches may accelerate rescue, particularly for lone workers or personnel operating in remote or high‐risk environments. In this context, wearables should not be viewed solely as monitoring devices, but as part of broader prevention strategies that combine real‐time feedback, environmental sensing, and organizational safety measures (V. Patel et al. 2022).

Climate change has increased the relevance of thermoregulatory monitoring during exercise (Kenney et al. 2022), particularly in physically active or exercising individuals, in whom metabolic heat production imposes substantial additional load on thermoregulatory homeostasis (Périard et al. 2021). Consequently, monitoring core body temperature (Tcore) using wearables or ingestible telemetric pills may support the prevention of heat‐related medical complications.

In athletic populations and especially outside of the laboratory, telemetric ingestible pills are increasingly used to assess core body temperature and have increased our understanding of the kinetics of Tcore to a variety of environmental and exercise conditions such as athletes competing in World Athletics Championships in hot‐humid conditions (Racinais et al. 2022) or at a World Cup Sprint Triathlon in the heat (James et al. 2025). However, the use of telemetric ingestible pills is not without limitations, as there is some debate on when to insert the telemetric pill and regarding the effects of cool water ingestions (Wilkinson et al. 2008). Additional technological developments include wearables, which are worn by the athletes, for example, using straps or as smart patches. However, the reliability and validity of such devices still need improvement in order to be applied with confidence in practice of athletes (Goods et al. 2023; Verdel et al. 2021; Xu et al. 2025).

A further safety‐related application of wearable technologies concerns the monitoring of single and cumulative head acceleration events using instrumented mouthguards to support concussion protocols, monitor athlete performance, aid in injury prevention (Haughey et al. 2026) and during return‐to‐play decisions (Evans et al. 2025). World Rugby mandated in 2024 to use instrumented mouthguards in elite‐level competitions (Haughey et al. 2026) and it may be anticipated that due to technological advancements and lowered costs, instrumented mouthguards will become more widely accessible also to youth and amateur sports (Haughey et al. 2026). Instrumented mouthguards are also employed in other sports such as soccer to assess head kinematics to model brain injury risk (Barnes‐Wood et al. 2024).

### 1.2 Prominent Challenges and a Call for Interdisciplinary Research

Several challenges need to be overcome to further leverage the potential of wearables in ambulatory assessments or improvement of aspects of physical activity, sedentary behavior, training, and sleep procedures in different healthy and diseased populations, which holds great potential for researchers from different disciplines as well as for academia and industry collaboration.

To better understand the challenges faced by the ECSS community, the ECSS WT SIG was conducted among the ECSS community. The survey was approved by the ethical committee of Exercise Science & Training of the Faculty of Human Sciences of the University of Würzburg (EV2026/12‐1206). The aim was to identify key issues that the SIG can address to develop impactful resources for the ECSS community.

*N* = 61 (41 male, 20 female) responded to the survey (age 37.1 ± 9.9 years) and represented a range of countries, with the highest proportions coming from Germany (*n* = 8), Canada (*n* = 7), Belgium (*n* = 6), Switzerland (*n* = 5), and the United Kingdom (*n* = 5), followed by several other European, Asian, and American countries. The most commonly used wearable technologies included smartwatches or fitness trackers (*n* = 57), smartphones (*n* = 34), research‐grade accelerometers (*n* = 32), and pedometers (*n* = 9). Participants reported frequent assessments of physical activity parameters (*n* = 63), heart rate during exercise (*n* = 58), resting heart rate (*n* = 54), GPS‐related parameters (*n* = 42), heart rate variability at rest (*n* = 34), and sleep parameters (*n* = 33). The majority of respondents reported working in athlete training (*n* = 21) or physical activity assessment (*n* = 17), with additional fields including recovery assessment (*n* = 7), sensor and algorithm development (*n* = 7), patient training (*n* = 5), and other specialized domains such as cognitive neuroscience, rehabilitation, and safety monitoring.

Figure 1 shows median ratings of posed challenges with wearables. While in general, there was a high variability in provided ratings, the four main challenges identified by the audience of the WT SIG were: Cost of devices limiting sample size or study duration (median: 4, Interquartile Range (IQR): 2), standardization of output metrics across different wearable brands (median: 4, IQR: 2), merging of data obtained by wearables with other data sources for example laboratory measures, subjective measures (median: 4, IQR: 2), and merging of data of obtained by different Wearables (median:4, IQR:2).

**FIGURE 1:**
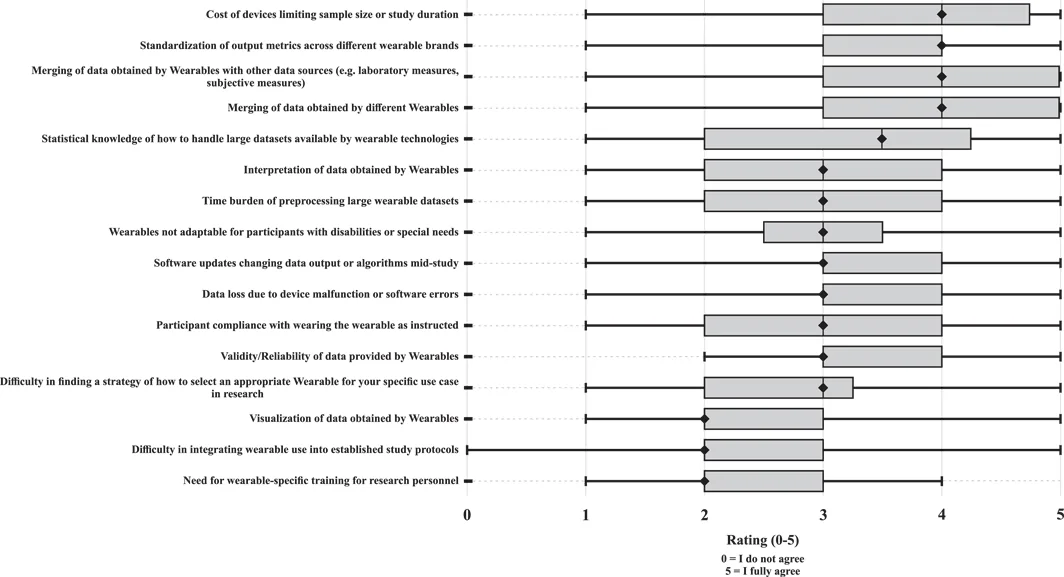
Survey results among the ECSS community; 0 indicating “do not agree”, 5 indicating “fully agree”.

Drawing on these findings, and informed by further discussions within the ECSS‐WT‐SIG regarding the positioning of the SIG, its members identified several key challenges that need to be addressed to advance wearable technology research in the contexts of physical activity, safety, athletic training, safety and sleep. These challenges, together with the proposed actions of the ECSS‐WT‐SIG to address them, have been grouped into four categories.

#### 1.2.1 Quality of Wearables

Although many consumer oriented wearables provide data relevant to healthcare, they lack regulatory oversight regarding, for example, reliability and validity (C. Doherty et al. 2024; Düking et al. 2016; Giurgiu et al. 2024). Consequently, many consumer oriented wearables have not undergone rigorous testing, raising concerns about the quality of the provided data and more research is needed in this area. Additionally, other aspects such as data security, privacy, ownership, and usability (next to other aspects) might be relevant when assessing the quality of a wearable (Robertson, Duthie, et al. 2023). To address this issue, establishing regulatory oversight and/or the establishment of an authority (potentially involving experts from within and outside academia) dedicated to evaluating the quality of wearables which aim to be used in ambulatory assessments or improvement of aspects of physical activity and sedentary behavior as well as exercise and training procedures in different healthy and diseased populations may be necessary. Such an authority (e.g., legitimized by professional associations from the scientific community or sports head organization) would enable practitioners to distinguish between high‐quality devices and those that do not perform adequately. It needs to be discussed which “authority” (e.g., IEEE, ISO, FDA) would be ideal for such quality assessment, especially since some sports governing bodies have introduced their own quality assessment programs (e.g., FIFA for EPTS systems (FIFA 2016), FIBA for equipment and software products (FIBA, n.d.), or World Rugby for instrumented mouthguards (World Rugby, n.d.)).

The ECSS WT SIG aims to contribute to this process for example, by conducting quality assessments of wearable technologies across different use cases, populations, and applied contexts. In addition, the ECSS WT SIG seeks to provide methodological guidance for researchers evaluating wearable devices and to collaborate with relevant scientific, professional, and regulatory bodies to develop frameworks, guidelines, and standards that improve the assessment, reporting, and overall quality of wearable technologies. Importantly, these activities should not be developed in isolation, but in close exchange with existing initiatives and networks in the field. For example, the Sports Tech Research Network (https://strn.co/) provides a platform for research‐driven innovation in sports technology, with a specific focus on the health, performance, and business dimensions of sport. Similarly, the Wearable Landscape initiative (https://wearable‐landscape.info/vision) aims to foster open‐science collaboration in wearable technologies and to advance the validation of wearables. By creating synergies with such initiatives, the ECSS WT SIG may help to reduce duplication of efforts, promote harmonized methodological standards, and support a more transparent, collaborative, and sustainable ecosystem for wearable technology research and application.

#### 1.2.2 Data Merging and Analysis

Promoting open Application Programming Interfaces and software development kits to connect different wearables (and other, e.g. laboratory‐based measures), which provide data in a standardized format would advance the field. This would allow researchers to connect and analyze the complex relationships between physical activity, sedentary behavior, and multiple bodily subsystems (such as cardiovascular, metabolic, musculoskeletal, mental) on a larger scale and/or more easily and would improve our understanding of the intricate interactions between different sub‐systems (e.g., physiological, psychological, environmental).

The ECSS WT SIG aims to contribute to improved data merging and analysis by providing evidence‐informed guidance to software developers, wearable manufacturers, researchers, and other relevant stakeholders. Specifically, the SIG seeks to support decisions regarding which data streams should be integrated in which scenario and application, how such data should be standardized and processed, and which analytical approaches may be appropriate in the given context. In addition, the SIG plans to support its members through online webinars and educational formats dedicated to key methodological and practical topics in wearable technology research. These webinars could address, for example, data preprocessing, sensor fusion, validation approaches, reporting standards, ethical and privacy considerations, and the interpretation of wearable‐derived metrics across different populations and settings. By offering such formats, the SIG aims to facilitate knowledge exchange among its members, strengthen methodological competence, and promote more consistent and transparent approaches to wearable technology assessment and application.

#### 1.2.3 Actionable Insights for Decision‐Making

Despite the valuable data collected by many wearables, translating wearable‐derived data into actionable insights remains challenging (Düking, Robertson, et al. 2024). Across many populations and scenarios, evidence remains insufficient on how decisions should be derived from wearable data, whether wearable‐based feedback improves outcomes, and whether it entails potential harms. Such decision‐making should be underpinned by foundational descriptive research (including population norms across cohorts and activity types), employing wearables which provide high quality data under relevant conditions and based on experimental studies employing wearables (part of) an intervention to evoke desired outcomes (e.g., improvement of step counts, improvement of sleep quality). Such research is often missing and overlooked in practice, partly because manufacturers' validity claims may appear convincing and discourage critical scrutiny. Future research should therefore develop strategies to translate wearable data into meaningful insights and evaluate whether these insights improve decision‐making while balancing benefits against potential negative effects.

The ECSS WT SIG aims to facilitate such research by performing studies within the laboratories and applied settings of its members, while also providing scientific guidance and support to other researchers working in this field. In addition, the SIG seeks to promote and coordinate collaborative systematic reviews and scoping reviews on key topics related to wearable technologies, such as device validity and reliability, methodological quality, reporting standards, and use‐case‐specific evidence across different populations and settings. These joint review activities could help to synthesize the rapidly growing literature, identify current knowledge gaps, and provide an evidence base for future recommendations, guidelines, and standards in wearable technology research and application.

#### 1.2.4 Improving Wearable Technology Literacy

Achieving desired outcomes while minimizing potential adverse outcomes will likely be at least partly dependent on how well the user is informed about selection (e.g., selecting devices which provide reliable and valid parameters) and correct application of wearables (e.g., correctness of parameter assessment and analysis) as well as understanding of provided metrics (e.g., contextualization of metrics). As the research as well as available wearables are constantly changing, there is an urgent need to develop educational resources to improve the literacy of different (end‐)users regarding wearables in ambulatory assessments to investigate physical activity, sedentary behavior, training, safety and sleep.

The ECSS WT SIG aims to generate, curate, and disseminate educational resources designed to enhance wearable technology literacy among sport scientists. By providing evidence‐informed guidance on device selection, data quality, parameter interpretation, and context‐specific application, the SIG seeks to support more informed and critical use of wearable technologies in research and applied sport science settings. In the long term, these activities could be complemented by the development of an online course or modular educational resource on wearable technology assessment, data integration, and data analysis. Such a course could translate methodological recommendations into accessible training materials, including examples of use‐case‐specific data merging, preprocessing workflows, validation procedures, and reporting standards. This would support capacity building among researchers and practitioners and help promote more consistent, transparent, and high‐quality approaches to wearable technology research and application.

The ECSS WT SIG acknowledges that in addition to the above‐mentioned challenges, other challenges such for example data privacy (C. Doherty et al. 2025), data ownership, accessibility to wearables and ethical issues (Arnold and Sade 2017; Robertson, Duthie, et al. 2023) are of relevance and require careful attention.

### 1.3 Leveraging Wearable Technology and Interdisciplinary Collaboration

While research shows that wearables can reveal new insights into physical activity, sedentary behavior, training, safety and sleep research, to overcome the above‐mentioned challenges, an interdisciplinary and transdisciplinary (e.g., involving stakeholders from industry, insurance companies, federations, end‐consumers) approach is likely required. Additionally, if these limitations can be overcome, interdisciplinary and transdisciplinary research applying wearables in real‐life scenarios in different populations and on a large scale can be facilitated. Today, many studies are conducted within a single or limited number of scientific disciplines and sometimes in laboratory settings. While such research is meaningful and has improved our understanding of physical activity, sedentary behavior, training, safety and sleep, we believe that in the future it will be increasingly valuable for researchers from many disciplines (e.g., exercise science, biomechanics, psychology, sociology) to collaborate in order to reveal interdependencies between parameters describing physical activity, sedentary behavior, training, safety and sleep.

For example, in the research field of exercise and training, intervention studies (employing different methodologies) are often performed comparing training method A versus training method B and/or a control group to derive evidence about the best training method in a certain (athletic) population and scenario. However, even for elite athletes, training time accounts for approximately 17% of waking time (depending on the specific sport discipline) (Fiskerstrand and Seiler 2004), and it was previously argued that other potential modulating factors occurring in the remaining time 83% of waking time (e.g., physical activity, nutrition, psycho‐social stress, environmental factors such as ambient temperature) as well as sleep should be monitored with (wearable) sensors (Sperlich and Holmberg 2017). Performing such studies with an interdisciplinary research team (e.g., exercise science, biomechanics, psychology, and sociology) would allow a clearer and more holistic view on adaptation in intervention studies within a specific population. Further, more available data would help to address the aforementioned challenges. With regards to the transdisciplinary approach, increased interest from the business sector or health insurance companies could support the scaling up.

The ECSS‐WT‐SIG anticipates that integrating different wearables which assess different parameters (e.g., related to physical activity, HRV) and contextual data (e.g., questionnaires, ambient sensors, GPS) into research will become increasingly important for generating comprehensive, interdisciplinary insights into different behaviors like physical activity, sedentary behavior, training, safety and sleep. Furthermore, this knowledge helps to achieve desired health and performance outcomes.

The Wearable Technologies Special Interest Group of the European Colleague of Sport Science invites interested individuals, research groups, industry partners, and NGOs to foster interdisciplinary and transdisciplinary collaboration between sports science, other academic disciplines, industry, governing bodies, end‐users and other stakeholders to conduct research about and with wearables in the field of physical activity, sedentary behavior, training, sleep and safety. This may include but is not limited to ambulatory assessment studies, randomized‐controlled trials, meta‐analysis and position statements in different populations and scenarios.

## Funding

The project was supported by the German Research Foundation through the Collaborative Research Center TRR265 (Grant No. 402170461), project C05 (to M.R.), by the ERA‐NET NEURON Project MASE (BMBF, Grant No. 01EW2404A to M.R.), and by the German Research Foundation (Grant No. 496846758 to M.G.).

## Conflicts of Interest

The authors declare no conflicts of interest.

## Data Availability

Data sharing is not applicable to this article as no datasets were generated or analyzed during the current study.
